# Healthcare Utilization Among United States Service Members with Combat-Related Lower Extremity Limb Salvage

**DOI:** 10.3390/healthcare13101164

**Published:** 2025-05-16

**Authors:** Susan L. Eskridge, Aidan McQuade, Benjamin Huang, Stephen M. Goldman, Christopher L. Dearth

**Affiliations:** 1 Leidos, San Diego, CA 92121, USA; 2 Department of Surgery, Uniformed Services University of the Health Sciences, Bethesda, MD 20814, USA; 3 Extremity Trauma and Amputation Center of Excellence, Defense Health Agency, Falls Church, VA 22042, USA

**Keywords:** military medicine, trauma, orthopedics, limb loss, reconstructive surgery

## Abstract

**Introduction**: This study assessed healthcare utilization in the first year after combat-related lower extremity injuries in 4275 U.S. Service members. Varying injury severity was hypothesized to correlate with different utilization patterns, with the limb salvage with secondary amputation (LS-SA) group expected to have the highest resource use. **Methods**: Data on inpatient admissions and outpatient visits were analyzed across four injury groups: primary amputation (PA), LS-SA, limb salvage with no amputation (LS-NA), and non-threatened limb trauma (NTLT). The LS-SA group had the highest mean total bed days and intensive care unit (ICU) days, with over 40% requiring four or more hospitalizations. The sample averaged 208.9 outpatient visits. Physical therapy, orthopedics, and social work had the highest clinic engagement. **Result**: Initial engagement in therapy clinics was high for PA and LS-SA but decreased for LS-NA and NTLT after the first quarter, while primary care engagement was more consistent. Physical therapy had the highest mean clinic utilization. Most initial inpatient admissions were at Landstuhl Regional Medical Center. PA and LS-SA received the majority of outpatient care at three Advanced Rehabilitation Centers, while care was more distributed for LS-NA and NTLT. This study underscores the substantial healthcare burden of combat-related lower extremity injuries, with the LS-SA group exhibiting the greatest resource utilization. **Conclusions**: The findings emphasize the need to optimize extremity trauma care across the Military Healthcare System as Service members with these injuries require significant healthcare resources, necessitating optimization of both care delivery and the military healthcare system.

## 1. Introduction

The conflicts in Iraq and Afghanistan resulted in a large volume of complex orthopedic injuries, with over half of all injuries affecting the extremities due to the increased use of improvised explosive devices and advancements in body armor. Improvements in prehospital care, including tourniquets, blood transfusions, and advanced transport, resulted in more critically injured combat casualties surviving to receive definitive surgical care [1,2,3].

Among these injuries, many threatened limb viability, necessitating multidisciplinary teams capable of delivering a high complexity of acute and chronic care [4,5,6,7,8,9,10]. In accordance with this high complexity of care, extremity trauma accounted for 65% of inpatient resource utilization within the Military Health System (MHS) during the height of the conflicts in Iraq and Afghanistan (October 2001 through January 2005) [11]. While the subset of these Service members who underwent limb loss during this period have been previously described [12], there is an unfortunate paucity of data on the clinical utilization pattern for those Service members who underwent limb salvage procedures [13,14]. Examples in the extant civilian trauma literature suggest that patients who enter the limb salvage clinical care pathway typically experience greater rates of rehospitalization and higher rates of complications [15,16].

This study aims to address this gap by investigating inpatient admissions of injured Service members (SM) and outpatient specialty clinic utilization, including location and timing of care for the limb salvage population compared to two other extremity trauma cohorts with varying degrees of injury severity and clinical care pathways: those who underwent a primary amputation and those with non-threatening limb trauma. The findings from this effort are critical in understanding where care was administered across the MHS for this extremity trauma cohort and will enable future efforts aimed at ensuring the MHS is fully equipped to provide excellent comprehensive care in the future. 

## 2. Methods

### 2.1. Data Sources and Study Sample

This study, approved by the Naval Health Research Center Institutional Review Board (Protocol #: NHRC.2003.0025, approved: 1 October 2003), consisted of a retrospective database review of all combat-related injuries to lower extremities between 1 January 2001 and 31 October 2015, with acute injury episodes documented in the Expeditionary Medical Encounter Database (EMED; NHRC, San Diego, CA, USA) [14]. Inclusion criteria included the requirement of inpatient medical records within two years of the date of injury accessible within the Military Health System Data Repository (MDR). Exclusion criteria included a maximum lower extremity Abbreviated Injury Scale (AIS) of one (i.e., minor trauma). Subsequently, an initial population of 4275 SM with combat-related lower extremity trauma was identified. The initial population was then stratified into primary amputations (PA; i.e., amputation occurring ≤14 days after injury), non-threatened limb trauma (NTLT), and limb salvage cohorts subdivided based on whether the patient went on to receive a secondary amputation (Limb Salvage; LS-SA) or not (i.e., Limb Salvage No Amputation; LS-NA) using a combination of medical codes that has previously been reported to be significantly associated with limb salvage [17,18]. Healthcare utilization patterns for the limb salvage cohorts were analyzed for one year following combat-related lower extremity injuries sustained during the study period, with the PA and NTLT cohorts serving as comparison groups. This one-year analysis focused on the period when Service members are known to experience the highest number of complications and aimed to minimize confounding factors due to medical retirements and transitions of care outside the Military Health System.

Data on inpatient admissions and outpatient clinic utilization within the first year after injury were obtained from the MDR. Military Treatment Facilities (MTFs) providing care were identified using the Defense Medical Information System Identifier. MTFs were reported when at least 1% of the total sample received either inpatient or outpatient care. Outpatient clinics of interest were identified based on prior reports [13] and authors’ expertise. Only clinics with at least one encounter for 5% of the total sample were included (Table 1).

### 2.2. Statistical Analysis

Total bed days and intensive care unit (ICU) bed days were compared across groups using analysis of variance (ANOVA) and Tukey’s method. The number of distinct inpatient admissions was reported with frequencies and percentages and compared across groups with chi-square analysis. The location of inpatient admission for the first hospitalization, last hospitalization, and total number of hospitalizations was reported for each injury group. All results are presented as mean ± standard deviation (SD). Outpatient clinic engagement and utilization were examined across all groups. Engagement was defined as having at least one encounter at an outpatient clinic or a specific clinic of interest. Engagement rates were reported for the entire year and for each quarter (0–3 months, 4–6 months, 7–9 months, 10–12 months). Chi-square analysis was used to compare engagement rates across injury groups. For patients with at least one encounter at a specific clinic, the mean ± SD of total visits was calculated and compared across injury groups with ANOVA and Tukey’s method, controlling for age, mechanism of injury, and polytrauma. The location of outpatient care was reported for each injury group.

## 3. Results

The study sample consisted of 4275 SM across four injury severity groups: PA (*n* = 885; 20.7%), LS-SA (*n* = 269; 6.3%), LS-NA (*n* = 1749; 41.2%), and NTLT (*n* = 1372; 32.1%). Inpatient admissions occurred in 4210 (98.5%) SM and were reported with total bed days, ICU days, and the categorized number of inpatient admissions (Table 2). SM with LS-SA had the highest mean number of total bed days (65.6 ± 56.7) and over 40% of those individuals required four or more hospitalizations. The LS-SA group also had higher mean ICU bed days than either the LS-NA or NTLT groups.

Within the first year after injury, the total study sample had an average of 208.9 visits to any outpatient clinic (range 2–1095). The PA group had a higher number of overall outpatient clinic visits (418.3 ± 175.2) compared to the other injury groups (LS-SA: 328.7 ± 163.2; LS-NA 153.3 ± 120.8; NTLT: 121.1 ± 108.2). The frequency and percentage of at least one encounter (i.e., clinic engagement) were examined (Table 3). The clinics with the highest engagement were physical therapy (*n* = 4158; 97.3%), followed by orthopedics (*n* = 3831; 89.6%), social work (*n* = 3388; 79.2%), occupational therapy (*n* = 3163; 74.0%), and primary care (*n* = 2854; 66.8%). The PA group had the highest clinic engagement at 8 out of the 13 clinics (psychiatric, psychology, occupational therapy, physical medicine, physical therapy, orthotic laboratory, neurology, and social work) compared to the other groups. The LS-SA group had the highest engagement in orthopedics (*n* = 261; 97.0%), pain management (*n* = 183; 68.0%), and plastic surgery clinics (*n* = 61; 22.7%), with the NTLT group having the highest engagement in mental health (*n* = 520; 37.9%) and primary care clinics (*n* = 949; 69.2%).

**Figure 1:**
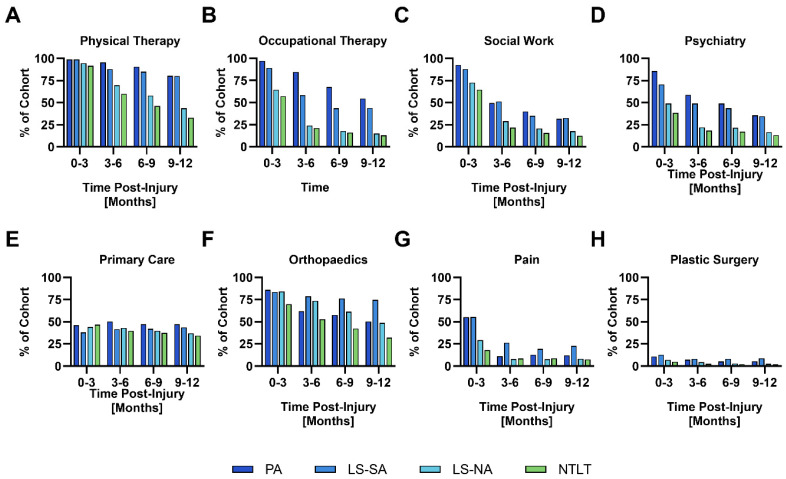
Outpatient clinic engagement for select clinics in the first year. The percentage of Service members with at least one visit to the clinic within each three-month period is depicted. These data are shown for different cohorts (PA, LS-SA, LS-NA, NTLT, as indicated by the color legend in the figure). Subpanels (**A**–**H**) detail this engagement for specific types of clinics or services: (**A**) Physical Therapy, (**B**) Occupational Therapy, (**C**) Social Work, (**D**) Psychiatry, (**E**) Primary Care, (**F**) Orthopaedics, (**G**) Pain, and (**H**) Plastic Surgery.

The patterns of clinic engagement by quarter intervals for representative clinics are displayed in Figure 1. The therapy-based clinics such as physical therapy (92–99%), occupational therapy (57–97%), social work (56–93%), and psychiatry (39–86%) have a substantial initial engagement frequency in the first quarter, especially in the PA and LS-SA groups, with SM engagement in 70% and 99% of at least one of the clinics, respectively (Figure 1A–D). After the first quarter, clinic engagement decreases—especially in the LS-NA and the NTLT groups—to a low of 12–13% in the last quarter for occupational therapy, social work, and psychiatry. In contrast, the primary care clinic exhibited a lower rate of engagement during the first quarter (38–46%) but experienced less of a decrease in engagement after the first quarter, with an engagement of 34–47% in months 10–12 (Figure 1E). For the LS-SA group, the rate of engagement at orthopedics (84%), pain (55%), and plastic surgery (13%) clinics was similar in the first quarter compared to the PA group (orthopedics 86%, pain 55%, plastic surgery 11%), but the rate of engagement did not diminish to the same degree as the PA group (last quarter engagement; orthopedics 75%, pain 23%, plastic surgery 9% for LS-SA, and orthopedics 50%, pain 12%, plastic surgery 5% for PA) (Figure 1F–H).

Means and SD were also examined to assess the most frequently utilized clinics for SM with at least one visit at the select clinic (Table 4). Similar to clinic engagement, physical therapy had the highest mean clinic utilization (58.9 ± 52.6), followed by occupational therapy (28.9 ± 37.9), psychiatry (14.9 ± 16.4), and orthopedics (14.8 ± 17.9). Clinic utilization across the study groups was compared while controlling for age, mechanism of injury, and polytrauma. Physical therapy, occupational therapy, and orthotics laboratory had higher mean utilization in the PA group compared to the other three groups, as did orthopedics, primary care, and social work compared to the LS-NA and NTLT groups. The LS-SA group had a higher mean utilization of mental health clinics compared to all other groups, and a higher utilization of psychology and pain management clinics compared to the LS-NA and NTLT groups.

The MTF locations for inpatient admissions (Table 5) and outpatient clinic encounters (Table 6) were examined. When comparing the location of the first and last admissions to inpatient facilities, as expected, over 95% of first admissions for all injury groups occurred at Landstuhl Regional Medical Center (LRMC). For the last admission, a similar trend is seen in the inpatient facilities as the outpatient facilities for the LS-NA and NTLT groups, with over 20% of last admissions at a wide range of facilities with less than one percent admissions overall. Most of the outpatient encounters occurred at one of the Advanced Rehabilitation Centers (ARC): Walter Reed Army/National Military Medical Center, Brooke Army Medical Center, and Naval Medical Center San Diego. When severity groups are compared, PA and LS-SA received over 90% (98% and 90%, respectively) of their care at one of those three facilities, whereas care was more distributed for the LS-NA and NTLT groups, with only 64% and 54% of care at those three clinics, respectively.

## 4. Discussion

This report investigated healthcare utilization within the first year after combat-related lower extremity trauma, focusing on four injury severity groups. As expected, the PA cohort utilized high levels of both inpatient and outpatient services [13]. However, this study highlights a crucial point: significant healthcare utilization across the entire spectrum of injury severity, not just the most severe, and a large number of MTFs providing care. This finding is particularly noteworthy because past research has primarily focused on programs for specialized populations at ARCs [4,5,6,7]. These studies often lack comprehensive data for comparison across various injury severities at all treatment facilities or have examined a limited number of outpatient clinics within a smaller sample size [13,19].

The results highlight the substantial burden of care for individuals undergoing LS procedures. Nearly all patients (99.3%) in the LS-SA group required at least two hospitalizations, with 40% requiring four or more. Similarly, the PA group had a 99.7% rate of two or more hospital admissions, while the LS-NA group still had a high rate (91.2%). These hospitalization rates surpass those reported for civilian patients with severe lower extremity injuries, regardless of limb salvage or amputation [20]. This discrepancy likely stems, in part, from the complex process of transferring combat-wounded patients from the battlefield to stateside hospitals, often involving consecutive care transfers. Consequently, this process can lead to multiple documented hospital admissions for those with severe injuries. However, the specific nature of combat injuries may also contribute to the higher admission rates. Blast injuries and high-energy mechanisms, more prevalent in combat, often result in more complex and severe injury patterns compared to the non-blast, lower-energy mechanisms typically seen in civilian populations [21,22].

The LS-SA group required the highest average inpatient stay (65.6 days), followed by the PA group (average: 56.2 days) (Table 1). The average inpatient stay across all groups was 33.9 days. A previous study by Mullenix et al. using civilian data from the National Trauma Data Bank reported an average of 16.9 inpatient days for popliteal artery injuries, a condition often requiring limb salvage or amputation [23]. Similarly, Mackenzie et al. found an average of 17.8 inpatient days during the initial hospitalization for lower extremity injuries [20]. Our findings show a significantly higher average inpatient stay for combat casualties compared to civilian data, despite a lower average ICU utilization (3.0 days across all groups). While all groups had higher average inpatient stays compared to civilian studies, only the amputation groups (PA and LS-SA) had similar ICU utilization.

Several explanations may account for this discrepancy. Combat injuries often involve concomitant injuries, potentially requiring longer hospital stays. However, one might expect this to translate to increased ICU stays as well. Another possibility is that the MHS, being more familiar with these types of injuries, may transition patients from ICU care to standard inpatient care faster than civilian institutions. Additionally, the military health system may support longer hospital stays for rehabilitation purposes, unlike the civilian sector, which is driven by cost constraints and insurance limitations. Finally, logistical concerns in the military setting, such as medical evacuation (MEDEVAC) coordination or limited patient housing near receiving hospitals (especially overseas), might contribute to extended inpatient stays. Further investigation is warranted to understand the multifactorial nature of this discrepancy.

### 4.1. Physical and Occupational Therapy Service Utilization

The study population exhibited significant outpatient clinic use across various healthcare domains. Within the first year, the average number of outpatient physical therapy (PT) visits for the PA, LS-SA, LS-NA, and NTLT groups were 120.4, 90.2, 41.7, and 32.8, respectively (Table 2). This contrasts with reports on civilian populations, which reported an average of 34.2 PT or occupational therapy (OT) visits over two years for patients undergoing LS procedures [20].

Similarly, the same study reported an average of 38.2 PT or OT visits for amputations over two years. In addition to increased PT utilization, the study population also demonstrated substantial use of outpatient surgical clinics (orthopedics and plastic surgery). This finding aligns with the higher rates of acute and long-term complications observed in this population.

Several factors might explain this increased outpatient utilization. More severe injuries due to blast mechanisms or concurrent combat injuries could contribute. Alternatively, systemic factors such as differences in clinical practice guidelines or universal access to care for Service members might play a role. This universal access may eliminate financial barriers present in non-universal healthcare systems.

Civilian care is often limited by cost and insurance restrictions. For example, Medicaid limitations on PT visits and requirements for prior authorizations, re-evaluations, and other hurdles may restrict access to care. Medicare has visit limitations for outpatient counseling, while private insurance may impose caps on PT duration and limitations on what is considered medically necessary. These limitations are absent in the military health system, potentially encouraging greater PT utilization.

Castillo et al. reported a significant proportion of civilian patients with severe lower extremity trauma not receiving PT despite a perceived need [24]. The military health system, by reducing barriers to care, might be addressing this disparity and leading to increased PT utilization compared to civilian counterparts.

### 4.2. Mental Health Service Utilization

All four injury groups displayed significant utilization of mental health services, with over 60% of patients accessing psychiatric care at some point following injury. Utilization rates decreased with decreasing injury severity (PA: 89.9%, LS-SA: 79.2%, LS-NA: 54.6%, NTLT: 44.8%). Notably, a recent report exhibited similar rates of post-traumatic stress disorder (PTSD) across all groups, with 58% of the LS-NA group experiencing at least one mental health issue [25]. Combat injuries are known risk factors for PTSD, and their prevalence has been documented in patients with combat-related lower extremity trauma [26,27]. Importantly, PTSD and depression following severe lower extremity injury are associated with poorer functional outcomes and perceived health, regardless of injury severity, baseline function, or degree of physical recovery [28,29]. Fortunately, diligent outpatient therapy can improve PTSD and its commonly accompanying depressive symptoms [30]. This trend might be reflected by the decline in mental health service utilization over time (Figure 1).

Previous research in both military and civilian settings has investigated the long-term utilization of mental health services following lower extremity trauma. Evidence suggests persistence of depressive symptoms up to two years post-injury and beyond [27,31]. Considering these findings, mental health services likely remain a heavily utilized domain of care for Service members recovering from lower extremity trauma, although the extent of this usage requires further investigation.

### 4.3. Peer Support Networks

Peer support plays a significant role in the recovery process, particularly for amputees who experience significant lifestyle changes due to limb loss. National organizations like the Amputee Coalition have established networks connecting recent amputees with individuals who have successfully adjusted to life after amputation. Sharing similar injuries, stories, and challenges fosters a valuable source of emotional support tailored to the unique experiences of limb loss. Many care centers emphasize these peer support groups, providing not only emotional support but also promoting functional recovery. The Amputee Coalition, among others, advocates for and offers support to individuals transitioning to and using prosthetics. Several studies highlight the importance of these peer groups and their benefits for patients’ mental and physical health [32,33,34,35].

Unfortunately, a similarly well-organized peer support network is lacking for those who undergo limb salvage procedures despite having nearly identical functional outcomes to amputees two years post-injury [36]. While our study did not focus on functional outcomes, the results demonstrate significant healthcare utilization across the first year post-injury for those who underwent limb salvage procedures, with or without eventual amputation. This high utilization is evident in clinics addressing functional support (orthopedics, physical therapy, occupational therapy) as well as those promoting emotional support (psychiatry, psychology, social work). Based on prior evidence and our findings, facilitating peer support networks for limb salvage patients may be beneficial, considering the documented challenges, functional deficits, and high care burden this group experiences. Fostering these connections could potentially improve mental and physical health outcomes, mirroring the positive impacts observed in the amputee community.

### 4.4. Distribution of Care for Complex Extremity Trauma Patients

Landstuhl Regional Medical Center typically serves as the initial MTF for combat-wounded service members (SMs) outside the combat zone. In this study, over 95% of first inpatient admissions for the entire cohort occurred at LRMC. Subsequent admissions for patients with severe injuries (PA and LS-SA groups) most frequently occurred at designated ARCs. Notably, over 97% of the PA group and over 85% of the LS-SA group received their last inpatient admission at an ARC facility. Conversely, patients with less severe injuries (LS-NA and NTLT groups) received care predominantly at non-ARC MTFs after their initial admission, with less than 50% of their last inpatient admissions occurring at ARCs. Similar trends were observed for outpatient care across these groups. These findings highlight the importance of continually evaluating the capabilities to care for patients with severe extremity trauma across the MHS, including at non-ARC MTFs. While the majority of patients treated at these facilities belong to the LS-NA and NTLT groups (assumed to have lower injury severity), our data demonstrate that they still require extensive inpatient and outpatient care. One potential solution could involve temporary transfers of patients with severe lower extremity trauma from non-ARC centers to ARCs for a portion or all of their care. However, such transfers might be resource-intensive and require careful consideration of cost implications. Logistical hurdles could also pose challenges to this approach, including family needs, transportation, military accountability, and housing availability. Furthermore, the factors determining patient disposition to ARC or non-ARC facilities warrant investigation. The current system might be placing patients requiring extensive support in hospital systems less equipped to provide the necessary level of care for recovery and rehabilitation. The potential clinical implications of these patient distribution patterns remain unclear and deserve further study to ensure optimal care for injured SMs.

### 4.5. Follow-Up and Limitations

Examining healthcare utilization through military medical databases is a unique contribution of this work. Previous research has documented the potential for combined care from the Department of Defense (DoD) and Veterans Affairs (VA) within the first year post-injury for SMs with limb loss. To capture the complete spectrum of care received by these patients, future research should include care provided by the VA healthcare system.

## 5. Conclusions

This analysis characterizes the substantial utilization of inpatient and outpatient care by Service members following combat-related lower extremity trauma. It notably includes the historically underreported limb salvage population and uniquely highlights how utilization varies with ultimate limb retention outcomes within this cohort. Among the groups studied, individuals who underwent limb salvage procedures and subsequently required amputation (LS-SA) demonstrated the highest resource utilization for both inpatient and outpatient care. Their care also extended longer within the first year post-injury compared to other injury groups. The limb salvage with no subsequent amputation (LS-NA) and no-trauma limb loss (NTLT) groups also required significant rehabilitative care delivered across various stateside military treatment facilities. These findings provide critical information for planning and optimizing acute and rehabilitative care for combat-wounded SMs, ensuring they continue to receive excellent and comprehensive care.

## Figures and Tables

**Table 1:** Military medical treatment facilities and outpatient clinics meeting inclusion threshold.

Military Medical Treatment Facilities
Blanchfield Army Community Hospital
Brooke Army Medical Center ^#^
Eisenhower Army Medical Center
Landstuhl Regional Medical Center
Madigan Army Medical Center
Naval Hospital Camp Lejeune
Naval Medical Center San Diego ^#^
Walter Reed Army/National Military Medical Center ^#^
Womack Army Medical Center-Ft Bragg
**Outpatient Clinics**
Mental Health
Neurology
Occupational Therapy
Orthopedics
Orthotic Laboratory *
Pain Management
Physical Medicine
Physical Therapy
Plastic Surgery
Primary Care
Psychiatry
Psychology
Social Work

Notes: * Indicates the code was only active up until 2014. ^#^ Indicates an Advanced Rehabilitation Center (ARC).

**Table 2:** Inpatient utilization for admissions within first year after injury by limb injury cohort.

Inpatient Utilization	PA *n* = 882		LS-SA *n* = 269		LS-NA *n* = 1716		NTLT *n* = 1343		*p* Value
	Mean	SD	Mean	SD	Mean	SD	Mean	SD	
Inpatient bed days ^1–6^	56.2	37.1	65.6	56.7	28.2	26.2	20.3	26.6	<0.001
ICU bed days ^2–5^	6.3	12.4	5.3	14.3	2.0	5.6	1.7	5.8	<0.001
Hospitalizations	*f*	%	*f*	%	*f*	%	*f*	%	<0.001
1	3	0.3	2	0.7	151	8.8	276	20.6	
2	318	36.0	60	22.3	893	52.0	708	52.7	
3	290	32.9	98	36.4	426	24.8	230	16.4	
≥4	271	30.7	109	40.5	246	14.3	139	10.3	

**Note**: Polytrauma defined as two AIS regions >2. *p* value for multivariate models adjusted by age, polytrauma (yes vs. no), and mechanism of injury (blast, gunshot wound, other). Tukey method used for mean comparisons: ^1^ Difference in means—PA and LS-SA, ^2^ Difference in means—PA and LS-NA, ^3^ Difference in means—PA and NTLT, ^4^ Difference in means—LS-SA and LS-NA, ^5^ Difference in means—LS-SA and NTLT, and ^6^ Difference in means—LS-NA and NTLT. Number of hospitalizations compared with chi-square.

**Table 3:** Frequency and percent outpatient clinic usage in first year after injury by limb injury cohort.

Outpatient Clinics	Total Sample (*n* = 4275)		PA (*n* = 885)		LS-SA (*n* = 269)		LS-NA (*n* = 1749)		NTLT (*n* = 1372)		*p* Value
	*f*	%	*f*	%	*f*	%	*f*	%	*f*	%	
Physical Therapy	4158.0	97.3	880.0	99.4	267.0	99.3	1706.0	97.5	1305.0	95.1	<0.001
Orthopedics	3831.0	89.6	837.0	94.6	261.0	97.0	1614.0	92.3	1119.0	81.6	<0.001
Social Work	3388.0	79.2	853.0	96.4	245.0	91.1	1339.0	76.6	951.0	69.3	<0.001
Occupational Therapy	3163.0	74.0	873.0	98.6	255.0	94.8	1202.0	68.7	833.0	60.7	<0.001
Primary Care	2854.0	66.8	572.0	64.6	167.0	62.1	1166.0	66.7	949.0	69.2	0.04
Psychiatry	2579.0	60.3	796.0	89.9	213.0	79.2	955.0	54.6	615.0	44.8	<0.001
Neurology	2447.0	57.2	569.0	64.3	166.0	61.7	940.0	53.7	772.0	56.3	<0.001
Physical Medicine	2186.0	51.1	869.0	98.2	222.0	82.5	653.0	37.3	442.0	32.2	<0.001
Orthotic Laboratory *	2005.0	46.9	716.0	80.9	187.0	69.5	730.0	41.7	372.0	27.1	<0.001
Pain Management	1708.0	39.9	544.0	61.5	183.0	68.0	626.0	35.8	355.0	25.9	<0.001
Mental Health	1521.0	35.6	280.0	31.6	99.0	36.8	622.0	35.6	520.0	37.9	0.02
Psychology	1217.0	28.5	274.0	31.0	81.0	30.1	492.0	28.1	370.0	27.0	0.2
Plastic Surgery	508.0	11.9	165.0	18.6	61.0	22.7	181.0	10.3	101.0	7.4	<0.001

Note: Frequency and percent of at least one encounter in each clinic. * Indicates active code to 2014 only. p value for chi-square comparison across all groups.

**Table 4:** Mean outpatient clinic visits in first year after injury by limb injury cohort.

Outpatient Clinics	Total Sample		PA		LS-SA		LS-NA		NTLT		*p* Value ^
	Mean	SD	Mean	SD	Mean	SD	Mean	SD	Mean	SD	
Physical Therapy ^1–6^	58.9	52.6	120.4	43.7	90.2	50.0	41.7	38.5	32.8	35.7	<0.001
Occupational Therapy ^1–5^	28.9	37.9	57.3	49.4	29.5	29.8	17.0	23.9	16.1	24.2	<0.001
Psychiatric ^2–5^	14.9	16.4	20.4	16.9	21.1	20.3	11.5	14.1	10.9	14.8	<0.001
Orthopedics ^2–6^	14.8	17.9	25.7	26.8	22.4	20.6	12.3	12.0	8.6	9.9	<0.001
Orthotic Laboratory *^,1–5^	13.6	18.0	30.0	19.8	14.2	13.3	2.8	2.8	2.7	3.3	<0.001
Primary Care ^2–6^	13.0	15.6	18.6	18.6	17.5	18.6	11.9	14.5	10.1	13.2	<0.001
Social Work ^2–6^	12.6	13.3	19.2	15.6	18.5	16.8	10.1	10.6	8.6	10.5	<0.001
Physical Medicine ^1–5^	12.1	12.8	20.1	13.3	12.5	12.1	5.9	8.2	5.3	7.3	<0.001
Pain Management ^2–5^	8.0	9.3	8.9	9.6	10.9	11.2	7.0	8.9	6.6	8.1	<0.001
Mental Health ^1,4,5^	7.1	11.4	6.5	6.7	11.5	20.6	6.3	8.0	7.6	14.0	<0.001
Plastic Surgery	5.5	7.5	4.5	7.0	6.8	9.1	6.3	8.5	4.8	4.9	0.19
Psychology	5.2	6.9	6.0	8.5	5.8	6.9	5.0	6.3	4.7	6.1	0.05
Neurology	3.4	5.3	4.0	5.2	3.8	6.0	3.2	5.7	3.3	4.8	0.28

Note: Mean/standard deviation for clinics include Service members who had at least one clinic visit. Subject numbers will change for each clinic. ^ *p* value for multivariate model comparison across 4 groups adjusted by age, polytrauma (yes vs. no), mechanism of injury (blast, gunshot wound, other). Polytrauma defined as two AIS regions >2. * active code to 2014 only. Tukey method used for mean comparisons between each group. ^1^ Difference in means—PA and LS-SA. ^2^ Difference in means—PA and LS-NA. ^3^ Difference in means—PA and Limb trauma. ^4^ Difference in means—LS-SA and LS-NA. ^5^ Difference in means—LS-SA and Limb trauma. ^6^ Difference in means—LS-NA and Limb trauma.

**Table 5:** Frequency and percent location of inpatient admissions in first year after injury by limb injury cohort.

Inpatient Admission Facility	PA *n* = 2824 (Total) *n* = 882 (First/Last Only)		LS-SA *n* = 971 (Total) *n* = 269 (First/Last Only)		LS-NA *n* = 4364 (Total) *n* = 1716 (First/Last Only)		NTLT *n* = 2983 (Total) *n* = 1343 (First/Last Only)	
	*f*	%	*f*	%	*f*	%	*f*	%
Walter Reed Army/National Military Medical Center								
Total	1297	45.9	395	40.7	1120	25.7	650	21.8
First admission	2	<1	2	<1	16	<1	16	1.2
Last admission	533	60.4	134	49.8	607	35.4	399	29.1
Landstuhl Regional Medical Center								
Total	881	31.2	267	27.5	1692	38.8	1305	43.7
First admission	880	99.8	265	98.5	1672	97.4	1284	95.6
Last admission	1	<1	2	<1	133	7.7	245	18.2
Brooke Army Medical Center								
Total	463	16.4	135	13.9	526	12	296	9.9
First admission	0	0	0	0	1	<1	5	<1
Last admission	246	27.9	62	23	289	16.8	174	13.0
Naval Medical Center San Diego								
Total	149	5.3	82	8.4	162	3.7	128	4.3
First admission	0	0	0	0	1	<1	4	<1
Last admission	84	9.5	35	13.0	98	5.7	77	5.7
Madigan Army Medical Center								
Total	2	<1	22	1.0	129	3.0	87	2.9
First admission	0	0	0	0	2	<1	5	<1
Last admission	1	<1	10	3.7	100	5.8	67	5.7
Womack Army Medical Center								
Total	2	<1	5	<1	138	3.2	82	2.7
First admission	0	0	0	0	4	<1	1	<1
Last admission	1	<1	1	<1	87	5.1	67	5.0
Eisenhower Army Medical Center								
Total	3	<1	6	<1	83	1.9	56	1.9
First admission	0	0	0	0	1	<1	2	<1
Last admission	3	<1	3	1.1	58	3.4	42	3.1
Other inpatient facilities (<1% individually)								
Total	27	1.0	59	6.1	515	11.8	379	12.7
First admission	0	0	2	<1	19	1.1	26	1.9
Last admission	13	1.5	22	8.2	344	20.0	272	20.2

Note: Military medical treatment facilities where at least 1% of the inpatient admissions occurred.

**Table 6:** Frequency and percent location of outpatient care in first year after injury by limb injury cohort.

Outpatient Location of Care	PA		LS-SA		LS-NA		NTLT	
	*n* = 268,807		*n* = 58,996		*n* = 167,900		*n* = 103,747	
	*f*	%	*f*	%	*f*	%	*f*	%
Walter Reed Army/National Military Medical Center	177,418	66.0	33,928	57.5	63,911	38.1	36,197	34.9
Brooke Army Medical Center	65,007	24.2	13,188	22.3	34,788	20.7	15,743	15.2
Naval Medical Center San Diego	21,036	7.8	6344	10.7	8013	4.8	4293	4.1
Landstuhl Regional Medical Center	2087	<1	601	1.0	3907	2.3	3433	3.3
Madigan Army Medical Center	156	<1	1006	1.7	5557	3.3	3239	3.1
Womack Army Medical Center-Ft Bragg	106	<1	274	<1	4996	3.0	3534	3.4
Naval Hospital Camp Lejeune	143	<1	89	<1	4228	2.5	3782	3.6
Blanchfield Army Community Hospital	96	<1	137	<1	4647	2.8	2719	2.6
Other outpatient facilities (<1% individually)	2758	1.0	3429	5.8	37,853	22.5	30,807	29.7

Notes: Military medical treatment facilities where at least 1% of the outpatient encounters occurred.

## Data Availability

The original contributions presented in this study are included in the article. Further inquiries can be directed to the corresponding author.

## References

[1] Owens B.D., Kragh J.F., Macaitis J., Svoboda S.J., Wenke J.C. (2007). Characterization of extremity wounds in Operation Iraqi Freedom and Operation Enduring Freedom. J. Orthop. Trauma.

[2] Owens B.D., Kragh J.F., Wenke J.C., Macaitis J., Wade C.E., Holcomb J.B. (2008). Combat wounds in operation Iraqi Freedom and operation Enduring Freedom. J. Trauma.

[3] Eskridge S.L., Macera C.A., Galarneau M.R., Holbrook T.L., Woodruff S.I., MacGregor A.J., Morton D.J., Shaffer R.A. (2012). Injuries from combat explosions in Iraq: Injury type, location, and severity. Injury.

[4] Goldberg C.K., Green B., Moore J., Wyatt M., Boulanger L., Belnap B., Harsch P., Donaldson D.S. (2009). Integrated musculoskeletal rehabilitation care at a comprehensive combat and complex casualty care program. J. Manip. Physiol. Ther..

[5] Fitzpatrick K.F., Pasquina P.F. (2010). Overview of the rehabilitation of the combat casualty. Mil. Med..

[6] Gordon W.T., Stannard J.P., Pasquina P.F., Archer K.R. (2012). Evolution of orthopaedic rehabilitation care. J. Am. Acad. Orthop. Surg..

[7] Rivera J.C., Pasquina P.F. (2016). Comprehensive Rehabilitation Following Combat Extremity Trauma: Evolution and Its Impact on Outcomes. J. Orthop. Trauma.

[8] Castillo R.C., Carlini A.R., Doukas W.C., Hayda R.A., Frisch H.M., Andersen R.C., D’Alleyrand J.-C., Mazurek M.T., Ficke J.R., Keeling J.J. (2021). Pain, depression, and posttraumatic stress disorder following major extremity trauma among United States military serving in Iraq and Afghanistan: Results from the military extremity trauma and amputation/limb salvage study. J. Orthop. Trauma.

[9] Melcer T., Walker J., Sechriest V.F., Bhatnagar V., Richard E., Perez K., Galarneau M. (2019). A retrospective comparison of five-year health outcomes following upper limb amputation and serious upper limb injury in the Iraq and Afghanistan conflicts. PM&R.

[10] Low E.E., Inkellis E., Morshed S. (2017). Complications and revision amputation following trauma-related lower limb loss. Injury.

[11] Masini B.D., Waterman S.M., Wenke J.C., Owens B.D., Hsu J.R., Ficke J.R. (2009). Resource utilization and disability outcome assessment of combat casualties from Operation Iraqi Freedom and Operation Enduring Freedom. J. Orthop. Trauma.

[12] Schulz R.N., Jannace K.C., Cooper D.B., Sparling T.L., Luken M.L., Pasquina P.F. (2024). Health Care Utilization After Major Limb Loss in Adults (18–64) Receiving Care in the Military Health System From 2001 to 2017. Arch. Phys. Med. Rehabil..

[13] Melcer T., Walker J., Bhatnagar V., Richard E. (2020). Clinic Use at the Departments of Defense and Veterans Affairs Following Combat Related Amputations. Mil. Med..

[14] Galarneau M.R., Hancock W.C., Konoske P., Melcer T., Vickers R.R., Walker G.J., Zouris J.M. (2006). The Navy-Marine Corps Combat Trauma Registry. Mil. Med..

[15] Harris A.M., Althausen P.L., Kellam J., Bosse M.J., Castillo R. (2009). Complications following limb-threatening lower extremity trauma. J. Orthop. Trauma.

[16] Busse J.W., Jacobs C.L., Swiontkowski M.F., Bosse M.J., Bhandari M. (2007). Complex limb salvage or early amputation for severe lower-limb injury: A meta-analysis of observational studies. J. Orthop. Trauma.

[17] Goldman S.M., Eskridge S.L., Franco S.R., Souza J.M., Tintle S.M., Dowd T.C., Alderete J., Potter B.K., Dearth C.L. (2023). A Data-Driven Method to Discriminate Limb Salvage from Other Combat-Related Extremity Trauma. J. Clin. Med..

[18] Goldman S.M., Eskridge S.L., Franco S.R., Dearth C.L. (2023). Demographics and Comorbidities of United States Service Members with Combat-Related Lower Extremity Limb Salvage. J. Clin. Med..

[19] Melcer T., Sechriest V.F., Walker J., Galarneau M. (2013). A comparison of health outcomes for combat amputee and limb salvage patients injured in Iraq and Afghanistan wars. J. Trauma Acute Care Surg..

[20] MacKenzie E.J., Jones A.S., Bosse M.J., Castillo R.C., Pollak A.N., Webb L.X., Swiontkowski M.F., Kellam J.F., Smith D.G., Sanders R.W. (2007). Health-care costs associated with amputation or reconstruction of a limb-threatening injury. J. Bone Jt. Surg..

[21] Wolf S.J., Bebarta V.S., Bonnett C.J., Pons P.T., Cantrill S.V. (2009). Blast injuries. Lancet.

[22] DePalma R.G., Burris D.G., Champion H.R., Hodgson M.J. (2005). Blast injuries. N. Engl. J. Med..

[23] Mullenix P.S., Steele S.R., Andersen C.A., Starnes B.W., Salim A., Martin M.J. (2006). Limb salvage and outcomes among patients with traumatic popliteal vascular injury: An analysis of the National Trauma Data Bank. J. Vasc. Surg..

[24] Castillo R.C., MacKenzie E.J., Webb L.X., Bosse M.J., Avery J. (2005). Use and perceived need of physical therapy following severe lower-extremity trauma. Arch. Phys. Med. Rehabil..

[25] Franco S.R., Eskridge S.L., Goldman S.M., Dearth C.L. (2025). Characterization of Secondary Health Conditions among United States Service Members with Combat-Related Lower Extremity Limb Salvage. J. Clin. Med..

[26] Richardson L.K., Frueh B.C., Acierno R. (2010). Prevalence estimates of combat-related post-traumatic stress disorder: Critical review. Aust. N. Z. J. Psychiatry.

[27] Doukas W.C., Hayda R.A., Frisch H.M., Andersen R.C., Mazurek M.T., Ficke J.R., Keeling J.J., Pasquina P.F., Wain H.J., Carlini A.R. (2013). The Military Extremity Trauma Amputation/Limb Salvage (METALS) study: Outcomes of amputation versus limb salvage following major lower-extremity trauma. J. Bone Jt. Surg..

[28] Holbrook T.L., Anderson J.P., Sieber W.J., Browner D., Hoyt D.B. (1999). Outcome after major trauma: 12-month and 18-month follow-up results from the Trauma Recovery Project. J. Trauma.

[29] Michaels A.J., Michaels C.E., Moon C.H., Smith J.S., Zimmerman M.A., Taheri P.A., Peterson C. (1999). Posttraumatic stress disorder after injury: Impact on general health outcome and early risk assessment. J. Trauma.

[30] Richardson J.D., Contractor A.A., Armour C., St Cyr K., Elhai J.D., Sareen J. (2014). Predictors of long-term treatment outcome in combat and peacekeeping veterans with military-related PTSD. J. Clin. Psychiatry.

[31] McCarthy M.L., MacKenzie E.J., Edwin D., Bosse M.J., Castillo R.C., Starr A. (2003). Psychological distress associated with severe lower-limb injury. J. Bone Jt. Surg..

[32] Reichmann J.P., Bartman K.R. (2018). An integrative review of peer support for patients undergoing major limb amputation. J. Vasc. Nurs..

[33] Keeves J., Hutchison A., D’Cruz K., Anderson S. (2023). Social and community participation following traumatic lower limb amputation: An exploratory qualitative study. Disabil. Rehabil..

[34] Murray C.D., Forshaw M.J. (2013). The experience of amputation and prosthesis use for adults: A metasynthesis. Disabil. Rehabil..

[35] Cain J.J., Ignaszewski D., Blymire C. (2021). Living Well After Amputation: Lessons in Innovation, Peer Support, and Health Policy. Tech. Orthop..

[36] Bosse M.J., MacKenzie E.J., Kellam J.F., Burgess A.R., Webb L.X., Swiontkowski M.F., Sanders R.W., Jones A.L., McAndrew M.P., Patterson B.M. (2002). An analysis of outcomes of reconstruction or amputation after leg-threatening injuries. N. Engl. J. Med..

